# Sling for the sling: a new technique for long-term correction of severe congenital ptosis

**DOI:** 10.1186/s12886-024-03371-3

**Published:** 2024-03-07

**Authors:** Ahmed N. Kotb, Noha M. Soliman, Ahmer Raza, Noran A. Nour, Hala K. Mattout

**Affiliations:** 1 Ophthalmology Department, Faculty of Medicine, Zagazig University, Zagazig, Egypt; 2 Institute of Ophthalmology, University College London, London, UK; 3 National Institute for Health Research Biomedical Research Centre for Ophthalmology at Moorfields Eye Hospital NHS Foundation Trust, London, UK; 4 Moorfields Eye Hospital NHS Foundation Trust, London, UK; 5 Faculty of Medicine, Ain Shams University, Cairo, Egypt

**Keywords:** Congenital ptosis, Silicon rods ptosis sling, Ethibond sutures, Sling for sling

## Abstract

**Introduction:**

Severe congenital ptosis poses a complex challenge for oculoplastic surgeons, requiring meticulous surgical intervention to restore eyelid function and improve aesthetic outcomes mainly by using frontalis sling approach. A crucial issue in frontalis sling surgeries is the sustainability of effect.

**Purpose:**

This retrospective study reports the outcomes of two surgical techniques for treating severe congenital ptosis in the paediatric age group: Silicon rods ptosis sling and a novel technique involving the use of Silicon rods with green braided polyester (Ethibond) sutures to secure the rods in place “sling for the sling”.

**Methods:**

The medical records of children who underwent frontalis suspension were reviewed in a retrospective fashion. We identified two groups; the first group (20 patients: 35 eyelids) had the traditional frontalis suspension surgery using silicone suspension set, the second group (14 patients: 25 eyelids) was operated using the new “sling for sling” technique. We used the postoperative marginal reflex distance-1 (MRD-1) as the primary outcome measure while the frequency of both wound related complications and recurrence were considered as secondary outcome measures. Post operative data were collected and compared after 1 month, 6 months, 12 months, and 18 months.

**Results:**

Preliminary results indicate promising outcomes for both techniques, with significant improvement in eyelid elevation observed in both groups. However, the novel technique using Silicon rods with Ethibond sutures demonstrated enhanced sustainability, leading to a more durable outcome with significantly less recurrence.

**Conclusion:**

This study highlights the potential benefits of the novel technique in treating severe congenital ptosis and introduces an innovative approach to Silicone rods fixation to achieve a long-term corrective effect.

**Supplementary Information:**

The online version contains supplementary material available at 10.1186/s12886-024-03371-3.

## Introduction

Congenital ptosis is a condition characterized by the drooping of one or both upper eyelids present at birth or within the first year of life which results in a narrow vertical palpebral fissure. It occurs due to the inadequate development of the levator palpebrae superioris (LPS) muscle [1]. Severe congenital ptosis can result in visual impairment and complications such as amblyopia, astigmatism, and ocular torticollis, which can necessitate early surgical intervention to preserve visual function [2].

The type of surgery performed depends upon levator function and ptosis severity. Tarsofrontalis sling is reserved for those with poor levator function (i.e. upper eyelid excursion less than 4 mm); it involves tethering the tarsal plate to the frontalis muscle with a ‘sling’ so that the brow and forehead movement are responsible for eyelid movement, bypassing the malfunctioning LPS [3].

Frontalis sling procedure is generally considered safe and effective, however the risk of lagophthalmos is significant in certain vulnerable patients namely those with poor Bell’s phenomenon, ocular myasthenia gravis, congenital fibrosis of extraocular muscles and other muscular dystrophies. Autogenous Fascia lata graft is commonly used as a sling for the surgery. However, harvesting the fascia lata requires an added surgical site, carrying the risk of possible donor site complications such as pain, impaired gait and persistent scarring [4]. Moreover, it is not recommended in children under 4 years of age limiting its wide use. All these concerns have led surgeons to seek alternative synthetic materials, including nonabsorbable suture materials (nylon, polypropylene, polyester), polytetrafluoroethylene and silicone rods [5]. Silicone possesses great elasticity which facilitates smooth and effective blinking motion, thus theoretically reducing risk of corneal complications. It also offers convenient adjustability in the event of any necessary revisions and allows for straightforward removal of the sling at a later date if deemed necessary. However, recurrence after silicone rods sling constituted a potential threat to long term patient satisfaction [3].

In this comparative study, we compare the outcomes of two surgical techniques of tarsofrontalis sling surgery– with and without the use of green braided polyester (Ethibond) sutures to fix the silicon rods in severe congenital ptosis patients.

## Patients and methods

This study was a retrospective single-centre analysis of patients with severe congenital ptosis who underwent surgical correction using two different techniques: the traditional Silicon rods ptosis sling and the novel technique using Silicon rods with Ethibond sutures for fixation. The study was conducted at the department of ophthalmology, faculty of medicine, Zagazig university, Zagazig, Egypt.

We reviewed the medical records of patients operated by both techniques in the period between December 2020 and December 2021. Criteria for participant selection were the following: confirmed diagnosis with unilateral or bilateral severe congenital ptosis with poor levator function (i.e., upper eyelid excursion is less than 4 mm), age below 12 years, no previous history of ptosis or any other eyelid surgeries, good Bell’s phenomenon and recorded completion of 18 months postoperative follow up. Patients with ocular myasthenia, poor Bell’s phenomenon, Marcus Gunn jaw winking and corneal hyposthesia were excluded from the study. The parents of all the participants provided an informed consent for their children to undergo the surgical procedures. The study was approved by the institutional review board of the faculty of medicine, Zagazig university, and the entire study protocol adhered to the principles outlined in the Declaration of Helsinki of 1964, as revised in 2013.

Data collected from patients’ records included demographic information, preoperative and postoperative marginal reflex distance (MRD1), levator function, follow up duration as well as postoperative complications.

### Surgical techniques

All surgeries were performed under general anaesthesia. Five Fox pentagon incisions were made as follows; A central incision located 1 cm above the eyebrow in line with the patient pupil, two stab incisions within the highest brow hairs in line with the medial and lateral canthi and the last two stab incisions were made after inserting a corneoscleral protector 3 mm from the upper eyelid margin in line with the temporal and nasal limbus. Blunt dissection was performed in the area of the central superior incision. The pre-attached needle of the silicone rods ptosis suspension set (Aurosling®, Aurolab, Tamil Nadu, India) was used to pass the silicone rod starting from the uppermost central incision and going downwards in the direction of the eyebrow then the eyelid incisions on one side (nasal or temporal) before returning upwards in the opposite direction emerging from the central incision again. The rods were secured by the sleeve provided with the suspension set and the sleeve position and rods tension were adjusted so that the eyelid height reaches the level of the superior limbus.

**Fig. 1:**
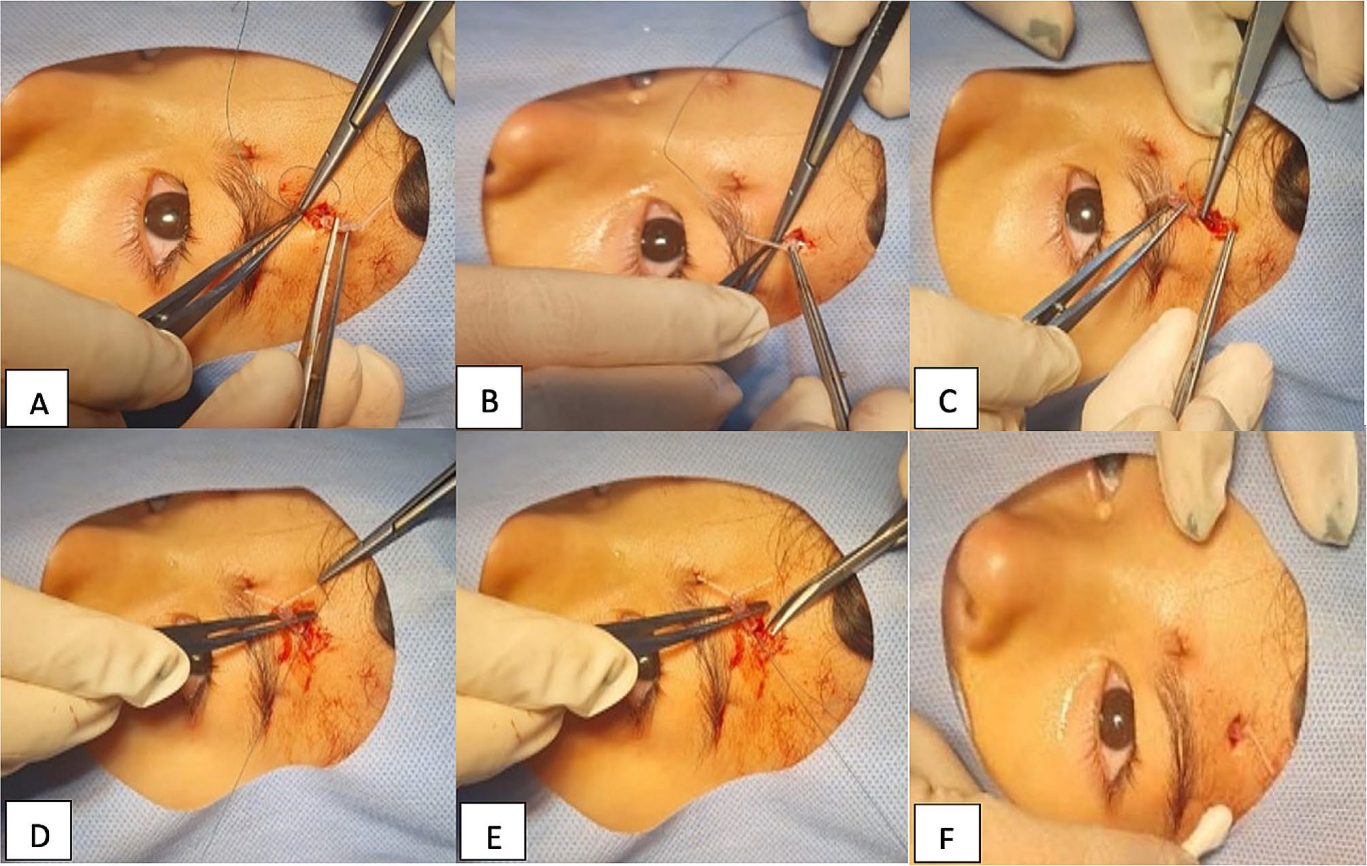
a: Illustration of the ‘sling for the sling’ surgical approach. **A**: first bite with 5/0 Ethibond in the muscular plane; **B**: passing the needle just below the sleeve of the silicon rod sling; **C**: second bite in the frontalis muscle; **D**: tightening the stitch around the silicon rod just below the sleeve; **E**: trimming of the stitches of Ethibond suture; **F**: reposition of the silicon rod then closure of the wound

**Fig. 2:**
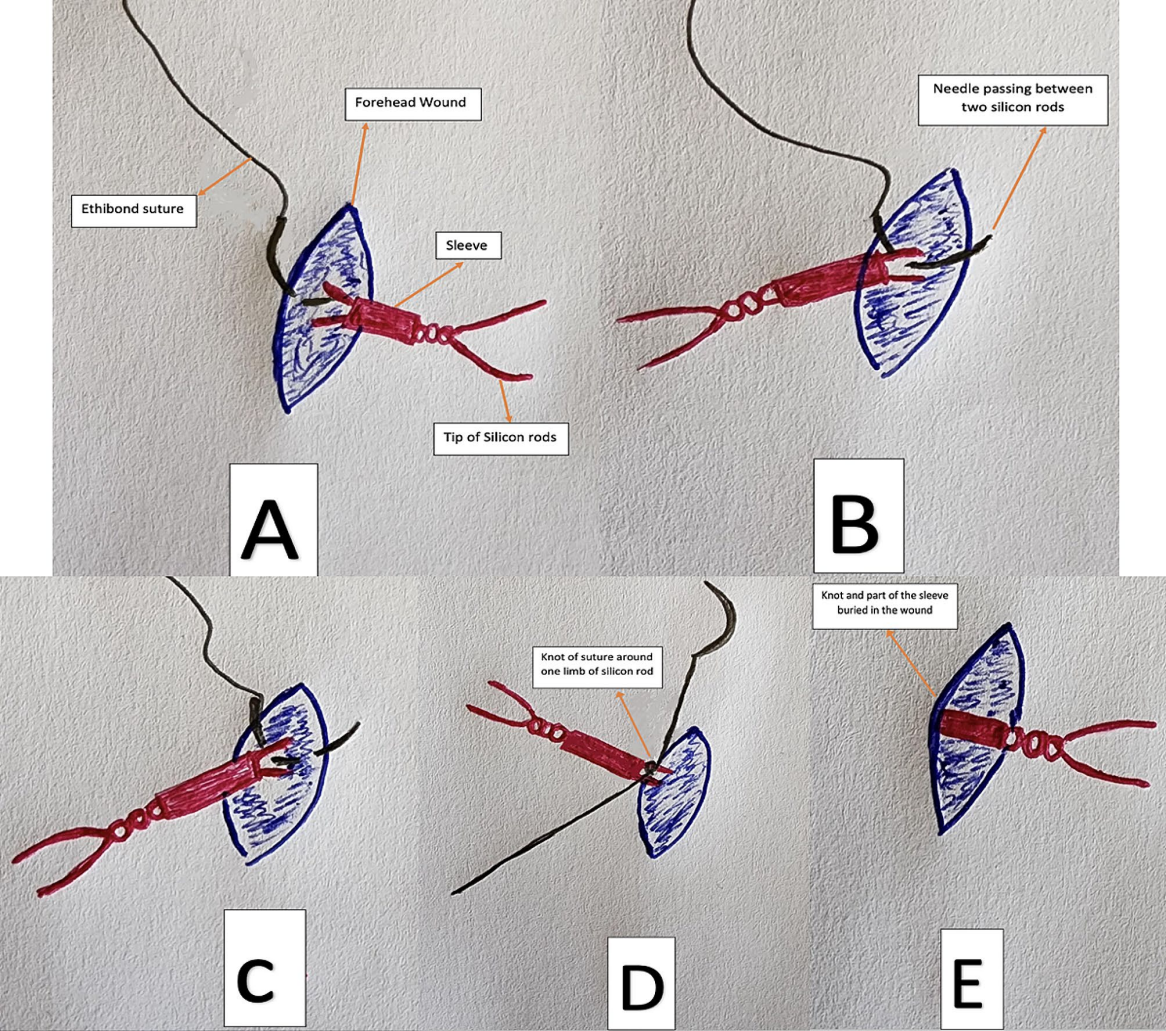
b: A schematic presentation of the suture technique. **A**: first bite with 5/0 Ethibond in the muscular plane; **B**: passing the needle just below the sleeve of the silicon rod sling; **C**: second bite in the muscular plane; **D**: tightening the stitch around the silicon rod just below the sleeve; **E**: trimming of the stitches of Ethibond suture and reposition of the silicon rod

In the first group of patients, no further fixation is added, and the excess silicone rods were trimmed, and the sleeve was buried in the central incision. For the second group, we implied the new technique of fixing the rods and sleeve with an additional sling like suture of Ethibond 5 − 0 “sling for sling technique”. The first bite was taken in the muscular (frontalis) plane then the suture was passed under the sleeve followed by another bite in the muscular plane. The knot was made over the silicone rod just below the sleeve fixing the rod in its place and preventing it from potential slippage. In both groups, the central uppermost wound was closed in two layers using 5 − 0 Vicryl sutures while the rest of the wounds were left unsutured as they rapidly close without significant scarring. A frost suture was applied in all patients to close the eyelids for the first 48 postoperative hours guarding against exposure keratopathy and removed if no significant exposure was noticed. Surgical steps are illustrated in Fig. 1a and a schematic presentation of the technique is presented in Fig. 2b.

Postoperatively, all patients were prescribed artificial tears, topical antibiotic ointment at bedtime and oral analgesics. Patients were advised to refrain from strenuous activities and to keep the operated eye elevated to minimize swelling.

Post operative data were collected and compared after 1 month, 6 months, 12 months, and 18 months. Timing of any recorded complication was also noted and included in our data analysis. The primary and main outcome measure assessed was the postoperative marginal reflex distance-1 (MRD-1). Based on postoperative MRD1 measured at the end of the first postoperative month, surgical correction was categorized as good (≥ 3 mm), fair (MRD is < 3 and ≥ 2)), or poor (MRD < 2 mm). Each eyelid was evaluated separately in bilateral cases.The secondary outcome measures mainly focused on postoperative complications, which encompassed the following aspects: recurrence which was defined as the conversion of the surgical correction result from good or fair to poor category with MRD < 2 mm and any wound-related problems such as infection, exposure of the sling material, or granuloma formation. Other complications were also taken into account including corneal complications namely exposure keratopathy and lid contour abnormalities as entropion, ectropion, lash ptosis and irregular eyelid contour.

### Statistical analysis

The quantitative variables distribution characteristics were tested by Shapiro–Wilk test; normally distributed data were described using mean and standard deviation (SD), while heterogenous non-normally distributed data were described by median (interquartile range (IQR)). Independent samples T-test was used for normally distributed quantitative variables, while Mann-Whitney test was used for heterogenous variables. Qualitative data analysis was done using Pearson’s chi squared test. For all tests, a P value of ˂ 0.05 was considered significant. Statistical Package of Social Services, version 29 (SPSS, IBM,) was used for data analysis.

## Results

A total of 60 eyelids of 34 patients were included in this study and according to the technique used for fixation of silicone rod sling, two groups of patients were identified. Group I which consisted of 35 eyelids of 20 patients operated with the traditional technique of silicone rod fixation using the sleeve only (15 bilateral, 5 unilateral), while group II included 25 eyelids of 14 patients who had received the new “sling for sling” technique (11 bilateral, 3 unilateral). The mean age of patients was 5 years (median 4.5; range 1–12 years). Both groups were comparable for baseline characteristics including age, gender, laterality, levator function and follow-up. Table 1 shows the baseline demographic data for both groups.

**Table 1:** The preoperative characteristics of the two studied groups

Variables	Group I *n* = 20 patients	Group II *n* = 14 patients	*P*
Age (years):			
Mean ± SD	5.4 ± 3.2	5 ± 3.1	0.8
Median (IQ range)	4.5 (1–12)	4.5 (1–12)	
Sex:			
Male	12 (60%)	8(57%)	0.7
Female	8 (40%)	6(43%)	
Upper eyelid excursion (mm)			
Mean ± SD	2.7 ± 0.9	2.6 ± 0.8	0.9
Range	3 (1–4)	3 (1–4)	
Laterality:			
Unilateral	5 (25%)	3(21.4%)	0.75
Bilateral	15 (75%)	11(78.6%)	
Follow up(months)			
Mean ± SD	21.3 ± 3	21.1 ± 3.1	0.9
Range	(18–28)	(18–27)	

Table 2 shows the preoperative MRD 1 and the final MRD1 measured at the last follow up visit in each group. Both groups show a significant increase in the postoperative MRD1 when compared to the preoperative MRD1. The postoperative MRD1 started to differ between both groups over the different follow up visits. By the last follow up visit; this difference showed a statistical significance (*p* = 0.04*) with a higher mean value in the sling for sling group.

**Table 2:** The changes in MRD1 measurements in the both groups

MRD1(mm) Mean ± SD Median (Range)	Group I (*n* = 35 eyelids)	Group II (*n* = 25 eyelids)	*P*
Pre-operative			
Mean ± SD	0.54 ± 0.7	0.4 ± 0.7	0.7
Median (range)	1(-1/1)	1(-1/1)	
1st postoperative month	3.07 ± 0.5	3.16 ± 0.62	0.5
	3(2.5-4)	3(2-4.5)	
6th postoperative month	2.4 ± 0.6	2.9 ± 0.5	0.1
	2.5 (1–3)	3 (2–4)	
12th postoperative month	2.2 ± 0.8	2.8 ± 0.5	0.06
	2.5(0–3)	3 (2–4)	
Last follow up visit	2.1 ± 0.8	2.8 ± 0.6	0.04*
	2.5(0–3)	3 (1–4)	
P1	< 0.0001*	< 0.0001*	
P2	0.005*	0.005*	

MRD1 margin reflex distance; P: difference among the three groups regarding preoperative MRD1 and postoperative MRD1 in each visit; P1: difference between preoperative MRD1 and the first month MRD1; P2: difference between MRD1 of the first month and the MRD1of the last follow up visit for each group; *statistically significant

Table 3 shows the postoperative complications recorded in both groups with statistically higher recurrence rate in the first group (*p* < 0.001*) while no detectable statistically significant difference existed regarding wound-related complications (*p* = 0.7). They included wound dehiscence, suture granuloma, or rod migration. Complications were managed effectively with conservative measures in most of the cases except for 2 patients (one in each group) who required sling removal due to its prolapse through the superior central incision.

**Table 3:** The postoperative main complications in the two studied groups

Complications	Group I (*n* = 35)	Group II (*n* = 25)	*P*
Granuloma [number (percentage)]	2(5.7%)	1(4%)	0.7
Infection [number (percentage)]	0	1(4%)	
Extrusion	1(2.8%)	0	
Total wound related complications	3(8.5%)	2 (8%)	
Recurrence [number (percentage)]	8 (23%)	2(8%)	< 0.001*
Mean time of recurrence (months)	16 ± 3.5	12.3 ± 2.5	

**Fig. 3:**
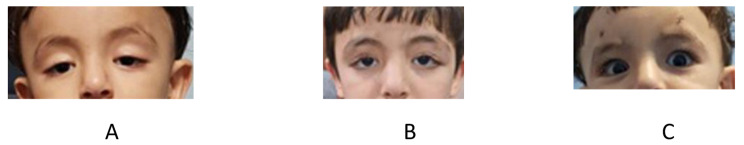
shows the preoperative **(A)**, the early postoperative (1 month) **(B)** and the final postoperative (18 months) **(C)** appearance of a patient belonging to the first group with moderate recurrence of ptosis

**Fig. 4:**
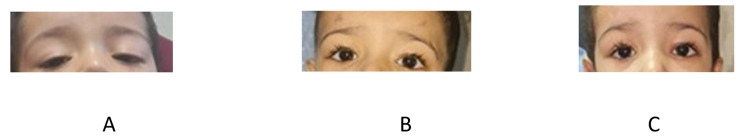
shows the preoperative **(A)**, the early postoperative (1 month) **(B)** and the late postoperative (18 months) **(C)** of a patient belonging to the second group showing the sustainability of the sling effect on application of the Ethibond sling fixation

**Fig. 5:**
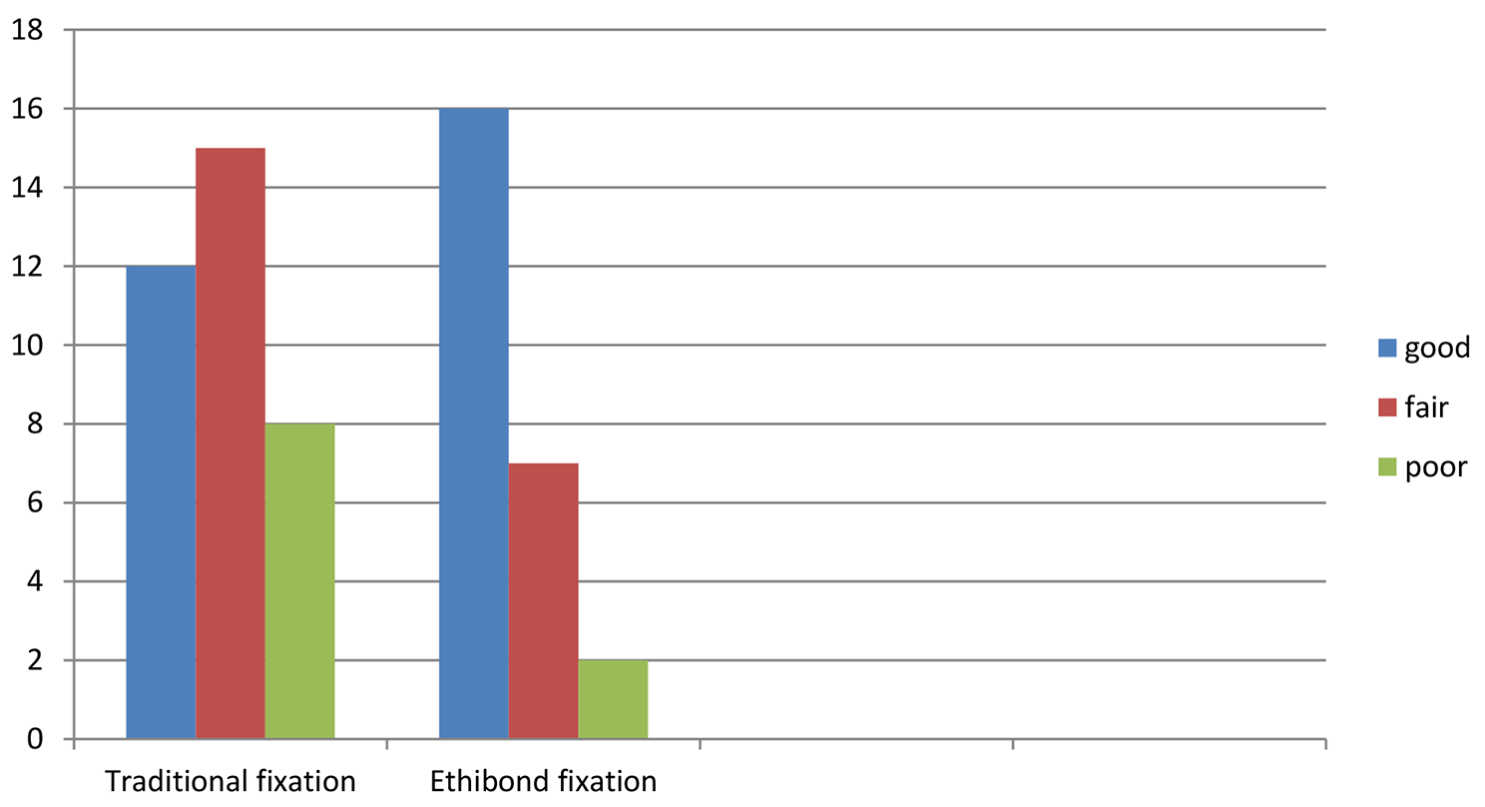
illustrates the outcome of both groups as recorded in the last follow up visit with statistically significant differences (*p* < 0.001)

Figure 3 shows the preoperative (A), the early postoperative (1 month) (B) and the final postoperative (18 months) (C) appearance of a patient belonging to the first group with moderate recurrence of ptosis. While Fig. 4 shows the preoperative (A), the early postoperative (1 month) (B) and the late postoperative (18 months) (C) of a patient belonging to the second group showing the sustainability of the sling effect on application of the Ethibond sling fixation. The outcome of both groups as recorded in the last follow up visit is illustrated in Fig. 5 with statistically significant differences (*p* < 0.001).

## Discussion

The management of severe congenital ptosis remains a complex and challenging task for oculoplastic surgeons, necessitating innovative surgical approaches for optimal outcomes. In this study, we conducted a comparative analysis of two distinct surgical techniques employed in the treatment of severe congenital ptosis: the traditional Silicon rods ptosis sling and a novel technique involving the use of Silicon rods with Ethibond sutures for fixation.

Our findings revealed that both the traditional Silicon rods ptosis sling and the novel technique using Silicon rods with Ethibond sutures were effective in achieving significant improvements in eyelid height and function among patients with severe congenital ptosis. These results are consistent with previous studies that have demonstrated the efficacy of the Silicon rods ptosis sling in correcting severe ptosis [6, 7, 8, 9]. The traditional technique’s reliability and consistent outcomes have made it a widely adopted approach for severe congenital ptosis.

However, the novel technique utilizing Silicon rods with Ethibond sutures introduces an innovative and promising approach to increase the durability of the correction. A significantly less incidence of recurrence was noticed in the second group that could be explained by the effect of the Ethibond suture which fixes the silicone rod to the frontalis muscle for a longer duration allowing more time for fibrosis to occur along the track of the sling material before losing its tensile stress and preventing its cheese-wiring into the muscular and subcutaneous tissue which are considered the main factors responsible for recurrence [10, 11].

The post-operative complications were evaluated within two groups. Group I (*n* = 23) displayed granulomas in 5.7% of cases, with no instances of infection. Conversely, a solitary case (2.8%) of extrusion was noted. The cumulative ensemble of wound-related complications was quantified at 8.5%. This rate is comparable to the rate encountered by Morris et al. (9%) [12] but higher than that reported by both Buttanri et al. [13] and Ben Simon et al. [14] who found no wound related complications when used silicone as a sling material.

In Group II (*n* = 25), granulomas and infections were noted in 4% of cases each and no cases of extrusion were noticed. The wound-related complications in Group II culminated at 8%, subtly mirroring Group I’s narrative. This rate agrees with the wound related complications rate reported by Wasserman et al. [15] (9.1%) and Bajaj et al. [16] (13%) who used Ethibond suture alone to perform the frontalis sling surgery.

Unexpectedly, we didn’t find a significant increase in the wound related complications in the ”sling for sling” group when Ethibond was added to silicone tube. This can be attributed to meticulous technique execution, including secure and robust fixation of the suture and sleeve to the underlying tissue, as well as the employment of a layered wound closure approach. These factors collectively contribute to the overall success and safety of using the silicon rods with Ethibond sutures for fixation technique.

Regarding the recurrence of complications, Group I displayed a recurrence rate of 23%, with a mean time of recurrence measured at 16 ± 3.5 months.This rate is similar to that reported by other studies using silicone alone e.g.Lee et al. [17] (29%) and Buttanri et al. [13] (23.75%).

In contrast, Group II exhibited a lower recurrence rate of 8%, with a mean time of recurrence recorded at 12.3 ± 2.5 months. The statistical analysis for recurrence rates demonstrated a highly significant p-value of less than 0.001, highlighting a substantial discrepancy in recurrence patterns between the two groups. This rate of recurrence is much lower than that reported by other studies that used other sling materials namely Ethibond suture as Bajaj et al. [16] (17%), Nylon suture as Hayashi et al. [18] (62%), fascia lata strip as Ben Simon et al. [14] (22%) or silicone tube alone as mentioned earlier. It is highly comparable to the low recurrence rate found when using autologous fascia lata [19, 20].

Tables S1 and S2 present the preoperative and postoperative characteristics, along with complications, for the traditional technique and the sling for sling surgical approach, respectively.

Despite the promising results, it is essential to consider the limitations of our study. The relatively small sample size and short-term follow-up limit the generalizability of the findings. Larger-scale, long-term studies with extended follow-up periods are necessary to validate the outcomes and assess the long-term stability and safety of the novel technique.

## Conclusion

Both the traditional Silicon rods ptosis sling and the novel technique using Silicon rods with Ethibond sutures offer viable surgical options for managing severe congenital ptosis. The traditional technique has a proven track record of reliability, while the novel approach introduces long term corrective effect with no associated increase in wound related complications. Further research with larger cohorts and longer follow-up periods is warranted to confirm these findings.

### Electronic supplementary material

Below is the link to the electronic supplementary material.

Supplementary Material 1

## Data Availability

All data generated or analysed during this study are included in this published article [and its supplementary information files].
