# Supplementary material for: Sling for the sling: a new technique for long-term correction of severe congenital ptosis
Source: BMC Ophthalmol. 2024 Mar 7;24:112. doi: 10.1186/s12886-024-03371-3 (PMC10921586; doi:10.1186/s12886-024-03371-3)
Supplement: Supplementary file 1 — Supplementary Material 1 [file 12886_2024_3371_MOESM1_ESM.docx]

Table S1: Preoperative and Postoperative Characteristics using the traditional technique with Postoperative Complications

| **Follow up** | **Age** | **Gender** | **Lid Excursion** | **Preop MRD1** | **Postop First MRD1** | **Postop last MRD1** | **Recurrence Time** | **Complication** |
| --- | --- | --- | --- | --- | --- | --- | --- | --- |
| 18 | 12 | m | 3 | 1 | 4 | 3 |  |  |
| 24 | 2 | f | 3 | 0 | 4 | 2 |  |  |
| 20 | 1 | m | 2 | -1 | 2 | 3 |  |  |
| 20 | 4 | m | 4 | 1 | 2.5 | 1 | 20 | EXTRUSION |
| 25 | 8 | m | 1 | 0 | 3 | 2 |  |  |
| 28 | 7 | f | 3 | 1 | 4 | 3 |  |  |
| 19 | 3 | m | 3 | 1 | 3 | 2 |  |  |
| 18 | 7 | m | 4 | 0 | 3 | 1 | 18 |  |
| 20 | 6 | m | 3 | 1 | 3.5 | 3 |  |  |
| 24 | 3 | f | 3 | 1 | 3.5 | 2 |  |  |
| 18 | 10 | f | 4 | 1 | 3.5 | 1 | 18 |  |
| 18 | 9 | m | 3 | 1 | 3 | 3 |  |  |
| 20 | 4 | f | 3 | 1 | 4 | 3 |  |  |
| 24 | 2 | m | 3 | 1 | 4 | 3 |  |  |
| 20 | 6 | f | 3 | 1 | 3 | 2 |  | GRANULOMA |
| 20 | 1 | m | 1 | -1 | 2.5 | 0 | 10 |  |
| 21 | 3 | m | 2 | 1 | 3 | 3 |  |  |
| 18 | 4 | m | 3 | 1 | 3 | 1 | 18 |  |
| 20 | 10 | f | 3 | 1 | 3 | 2.5 |  |  |
| 27 | 5 | f | 4 | 1 | 3.5 | 2.5 |  |  |
| 21.1 | 5.35 | 12m | 2 | -1 | 2.5 | 2 |  |  |
| 3.110255 | 3.216323 | 8f | 1 | 0 | 2.5 | 2 |  |  |
| 20 | 4.5 |  | 3 | 1 | 3 | 1 | 18 |  |
|  |  |  | 2 | 0 | 3 | 0 | 12 |  |
|  |  |  | 2 | 0 | 3 | 3 |  |  |
|  |  |  | 3 | 1 | 3 | 3 |  |  |
|  |  |  | 2 | -1 | 3 | 2 |  |  |
|  |  |  | 4 | 1 | 3 | 2 |  |  |
|  |  |  | 2 | 0 | 2.5 | 2.5 |  |  |
|  |  |  | 1 | 0 | 2.5 | 2.5 |  |  |
|  |  |  | 2 | 1 | 3 | 3 |  | GRANULOMA |
|  |  |  | 2 | 1 | 3 | 3 |  |  |
|  |  |  | 3 | 1 | 3 | 1 | 14 |  |
|  |  |  | 4 | 1 | 3 | 2.5 |  |  |
|  |  |  | 2 | -1 | 2.5 | 2.5 |  |  |
|  |  |  | 2.657143 | 0.485714 | 3.071429 | 2.142857 | 16 |  |
|  |  |  | 0.905631 | 0.742469 | 0.502096 | 0.88759 | 3.545621 |  |
|  |  |  | 3 | 1 | 3 | 2.5 | 18 |  |

| **Follow up** | **Age** | **Gender** | **Lid Excursion** | **Preop MRD1** | **Postop First MRD1** | **Postop Last MRD1** | **Complication** | **Recurrence Time** |
| --- | --- | --- | --- | --- | --- | --- | --- | --- |
| 22 | 5 | f | 4 | 1 | 3 | 3 |  |  |
| 19 | 1 | m | 1 | 0 | 4 | 2 |  |  |
| 20 | 2 | m | 2 | -1 | 4 | 3 |  |  |
| 22 | 3 | m | 2 | 0 | 3 | 3 |  |  |
| 18 | 5 | f | 3 | 0 | 2.5 | 2 |  |  |
| 22 | 7 | m | 4 | 0 | 2.5 | 2.5 |  |  |
| 20 | 2 | f | 3 | 1 | 4 | 1.5 | GRANULOMA | 12 |
| 18 | 4 | m | 3 | 1 | 3 | 2.5 |  |  |
| 18 | 3 | m | 2 | 1 | 3.5 | 3.5 |  |  |
| 19 | 6 | f | 3 | -1 | 3 | 3 |  |  |
| 22 | 10 | m | 4 | 0 | 2.5 | 3 |  |  |
| 21 | 12 | f | 4 | 1 | 4 | 4 |  |  |
| 18 | 6 | m | 3 | 1 | 3 | 3 |  |  |
| 18 | 4 | f | 1 | -1 | 3 | 3 |  |  |
|  |  |  | 3 | 1 | 3.5 | 3 |  |  |
|  |  |  | 2 | 1 | 3 | 3 |  |  |
|  |  |  | 2 | 1 | 2.5 | 2.5 |  |  |
|  |  |  | 3 | 0 | 3 | 3 |  |  |
| 19.78571 | 5 |  | 4 | 0 | 3 | 3 | INFECTION |  |
| 1.717716 | 3.08844 |  | 4 | 1 | 3 | 2.5 |  |  |
| 19.5 | 4.5 |  | 3 | 1 | 3 | 3 |  |  |
|  |  |  | 2 | 1 | 4 | 3.5 |  |  |
|  |  | 8m | 3 | 0 | 4.5 | 4 |  |  |
|  |  | 6f | 2 | 1 | 2.5 | 1 |  | 10 |
|  |  |  | 4 | 1 | 2 | 2 |  |  |
|  |  |  | 2.84 | 0.44 | 3.16 | 2.78 |  | 11 |
|  |  |  | 0.943398 | 0.711805 | 0.6245 | 0.6245 |  | 1.414214 |
|  |  |  | 3 | 1 | 3 | 3 |  | 11 |

Table S2: Preoperative and Postoperative Characteristics using the sling for sling technique with Postoperative Complications
